# Individuals with inflammatory breast cancer at higher risk of cancer spread to the brain

**Published:** 2022-11

**OCTOBER 10, 2022** - New research indicates that among individuals with breast cancer, those with a rare subtype called inflammatory breast cancer face a higher risk that their cancer will spread, or metastasize, to the brain. The study is published by Wiley online in CANCER, a peer-reviewed journal of the American Cancer Society.

Studies have demonstrated higher rates of brain metastases in patients with inflammatory breast cancer, but detailed information is lacking. To provide insights into the incidence and risk factors for brain metastases in this patient population, Laura E.G. Warren, MD, of the Dana-Farber Cancer Institute, and her colleagues analyzed data on 372 patients with stage III inflammatory breast cancer and 159 with stage IV inflammatory breast cancer.

Over a median follow-up of 5 years, the incidence of brain metastases at 1, 2, and 5 years was 5%, 9%, and 18% among patients who presented with stage III disease, and 17%, 30%, and 42% among those with stage IV disease. Patients with triple-negative breast cancer faced a particularly high risk, and when they did experience brain metastases, their survival time was shorter than those with hormone receptor–positive or HER2-positive breast cancer who experienced brain metastases. Higher risks of brain metastases were also seen in patients whose cancer had metastasized to other parts of the body besides the brain, especially when this occurred at a young age.

“The relatively high incidence of brain metastases seen in the study population highlights the need for future research on the potential role for surveillance brain imaging for high-risk patients. There is an open, phase II, single arm study at Dana-Farber Cancer Institute examining this question,” said Dr. Warren. “It also emphasizes the need to obtain brain imaging in patients with inflammatory breast cancer presenting with neurologic symptoms given the high incidence of brain metastases in this population.”

Most patients in this study who were diagnosed with brain metastases had neurologic symptoms, but because some patients may have undetected, asymptomatic brain metastases, the true incidence in patients with inflammatory breast cancer is likely even higher than what Dr. Warren and her colleagues observed.

An accompanying editorial notes that when considering whether to implement routine brain imaging tests in patients with inflammatory breast cancer, it will be important to determine whether earlier detection of brain metastases leads to improvements in both survival and quality of life.

***Link to Study: http://doi.wiley.com/10.1002/cncr.34441***

***Full citation:** “Incidence, characteristics, and management of central nervous system metastases in patients with inflammatory breast cancer.” Laura E.G. Warren, Samuel M. Niman, Marie C. Remolano, Jean M. Landry, Faina Nakhlis, Jennifer R. Bellon, Ayal A. Aizer, Nancy U. Lin, Sara M. Tolaney, Meredith M. Regan, Beth A. Overmoyer, and Filipa Lynce. CANCER; Published Online: October 10, 2022 (DOI: 10.1002/cncr.34441).*

*Copyright © 2019 The Cochrane Collaboration. Published by John Wiley & Sons, Ltd., reproduced with permission.*

